# LncRNA–miRNA interactions prediction based on meta‐path similarity and Gaussian kernel similarity

**DOI:** 10.1111/jcmm.18590

**Published:** 2024-09-30

**Authors:** Jingxuan Xie, Peng Xu, Ye Lin, Manyu Zheng, Jixuan Jia, Xinru Tan, Jianqiang Sun, Qi Zhao

**Affiliations:** ^1^ School of Computer Science and Software Engineering University of Science and Technology Liaoning Anshan China; ^2^ School of Electronic and Information Engineering University of Science and Technology Liaoning Anshan China; ^3^ College of Computer Science and Technology Jilin University Changchun China; ^4^ The First School of Medicine, School of Information and Engineering Wenzhou Medical University Wenzhou China; ^5^ School of Information Science and Engineering Linyi University Linyi China

**Keywords:** attention network, doc2vec, feature fusion, graph, lncRNA–miRNA interactions, meta‐path

## Abstract

Long non‐coding RNAs (lncRNAs) and microRNAs (miRNAs) are two typical types of non‐coding RNAs that interact and play important regulatory roles in many animal organisms. Exploring the unknown interactions between lncRNAs and miRNAs contributes to a better understanding of their functional involvement. Currently, studying the interactions between lncRNAs and miRNAs heavily relies on laborious biological experiments. Therefore, it is necessary to design a computational method for predicting lncRNA–miRNA interactions. In this work, we propose a method called MPGK‐LMI, which utilizes a graph attention network (GAT) to predict lncRNA–miRNA interactions in animals. First, we construct a meta‐path similarity matrix based on known lncRNA–miRNA interaction information. Then, we use GAT to aggregate the constructed meta‐path similarity matrix and the computed Gaussian kernel similarity matrix to update the feature matrix with neighbourhood information. Finally, a scoring module is used for prediction. By comparing with three state‐of‐the‐art algorithms, MPGK‐LMI achieves the best results in terms of performance, with AUC value of 0.9077, AUPR of 0.9327, ACC of 0.9080, F1‐score of 0.9143 and precision of 0.8739. These results validate the effectiveness and reliability of MPGK‐LMI. Additionally, we conduct detailed case studies to demonstrate the effectiveness and feasibility of our approach in practical applications. Through these empirical results, we gain deeper insights into the functional roles and mechanisms of lncRNA–miRNA interactions, providing significant breakthroughs and advancements in this field of research. In summary, our method not only outperforms others in terms of performance but also establishes its practicality and reliability in biological research through real‐case analysis, offering strong support and guidance for future studies and applications.

## 1 INTRODUCTION

Non‐coding RNA (ncRNA) is a category of RNA molecules that lack the encode proteins.^1^ Among them, lncRNA is a subclass of ncRNA typically characterized by a length of over 200 nucleotides,^2^ whereas miRNA is a subclass of ncRNA with a length of around 20 nucleotides.^3^ Recent research has revealed the extensive prospects of studying lncRNA–miRNA interactions. These intricate interactions are pivotal in finely coordinating and regulating diverse biological processes, disease diagnosis and treatment, and drug development.^4^ In particular, lncRNA–miRNA interactions in animals have been uncovered modulate gene expression at the level of post‐transcriptional regulation, contributing to the regulation of cell differentiation, development, metabolism, and various biological processes. For instance, in mice, the interaction between lncRNA GAS5 and miR‐21 plays a significant role in cardiovascular diseases. GAS5 binds to miR‐21, thereby reducing miR‐21's inhibitory effect on its target genes and regulating the growth and apoptosis of cardiac cells.^5^ In drosophila, the interaction between lncRNA roX2 and miR‐9a is involved in the regulation of the male sex determination factor sex‐lethal (SXL). By binding to miR‐9a, roX2 modulates miR‐9a's regulatory effect on SXL, thereby impacting the sex determination in fruit flies.^6^ Therefore, exploring unknown animal lncRNA–miRNA interactions not only reveals the complex regulatory networks within animal cells, helping us gain a better understanding of the functional manifestation of lncRNA and miRNA but it also offers new directions and valuable insights for advancing biomedical research on biological processes. Hence, delving into uncharted animal lncRNA–miRNA interactions not only unveils the intricate regulatory networks within animal cells, enhancing our comprehension of lncRNA and miRNA functional expression but also offers fresh perspectives and avenues for biomedical research into biological processes. A series of experimental techniques in biology, such as quantitative reverse transcription polymerase chain reaction,^7^ microarrays and high‐throughput sequencing,^8^ can be used to validate lncRNA–miRNA interactions. However, these experimental methods require significant human and material resources, and the validation process can be tedious and prone to higher chances of failure. To overcome these drawbacks, researchers have put forward computational and artificial intelligence methodologies for predicting lncRNA–miRNA interactions. These methods utilize big data analysis and machine learning algorithms, combined with publicly available bioinformatics databases, to forecast potential lncRNA–miRNA interactions. Such computational approaches save time, resources, and effort while increasing the reliability of experimental results.

Recently, the continuous advancement in computational capabilities has led to the widespread application of machine learning techniques, particularly deep learning methods, across various bioinformatics disciplines. These include the analysis of remote health monitoring,^9^, ^10^, ^11^ computational toxicology,^12^, ^13^, ^14^, ^15^ metabolite‐disease associations prediction,^16^, ^17^, ^18^ miRNA or lncRNA correlation studies,^19^, ^20^, ^21^ drug and microbial response studies,^22^, ^23^, ^24^ and the prediction of associations between circRNA and diseases.^25^, ^26^, ^27^ These investigations provide strong evidence for the continual improvement of computational models for lncRNA–miRNA interactions. For example, in 2020, Fan et al. proposed a heterogeneous graph inference method based on similarity network fusion (SNFHGILMI) to predict potential lncRNA–miRNA interactions.^27^ SNFHGILMI offers the advantage of capturing valuable information from individual networks and achieving enhanced performance when combining networks that lack an apparent linear relationship. In the same year, Liu et al. introduced a novel matrix factorization model called lncRNA–miRNA interactions prediction by logistic matrix factorization with neighbourhood regularized (LMFNRLMI) for predicting lncRNA–miRNA interactions.^28^ LMFNRLMI specifically employs known positive samples to discover potential associations in datasets lacking negative samples and has demonstrated reliable performance in cross‐validation experiments. However, both SNFHGILMI and LMFNRLMI are specifically designed for predicting potential human lncRNA–miRNA interactions. In 2021, Kang et al. designed a method based on dual‐path parallel integration and proposed a new integrated pruning protocol for predicting plant lncRNA–miRNA interactions.^29^ This approach, utilizing a decision model and a semi‐dynamic mechanism, enables the pruning of the base model without requiring a set for validation, thereby improving the accuracy of the model on the validation set. In 2022, Zhou et al. proposed Pmli‐TF for predicting miRNA–lncRNA interactions. Pmli‐TF follows a four‐step process: input embedding, positional encoding, multi‐head attention and max pooling.^30^ The multi‐head attention further improves the model's capacity to capture long‐range dependencies. Pmli‐TF demonstrates strong generalization capabilities. However, the primary focus of this study is on lncRNA–miRNA associations in plants. In the same year, Wang et al. proposed a method based on graph convolutional neural network and conditional random field (GCNCRF) for predicting human lncRNA–miRNA interactions.^31^ GCNCRF utilizes an attention mechanism to update node weights, allowing each node to redistribute its weights based on the differences with its neighbouring nodes, thereby making better use of the neighbourhood information and achieving higher prediction accuracy. Therefore, leveraging the neighbourhood information of nodes to its fullest extent is highly necessary. Subsequently, Zhuo et al. introduced a method that utilizes the concept of a bipartite graph to predict lncRNA–miRNA interactions in plant datasets.^32^ In their study, they first extracted attribute information and structure information as crucial features, which were subsequently processed for network embedding. To construct the loss function and facilitate parameter training, they employed techniques such as intra‐partition and inter‐partition proximity modelling. In 2023, Zhao et al. introduced the semantic embedded bipartite graph network (SEBGLMA) for predicting human lncRNA–miRNA associations.^33^ This method incorporates innovative feature extraction techniques by combining K‐mer segmentation, word2vec, Gaussian interaction profile, and graph convolution network. Subsequently, Wang et al. proposed a method called sequence pre trained graph neural network (SPGNN) for predicting animal miRNA–lncRNA interactions.^34^ Despite utilizing sequence pretraining to improve the prediction of miRNA–lncRNA interactions using graph neural networks and evaluating the performance of various pretraining methods, this approach does not fully capitalize on the information available in the node neighbourhoods.

Most existing methods for predicting lncRNA–miRNA interactions are primarily focused on humans or plants, with limited approaches available for predicting animal lncRNA–miRNA interactions. Therefore, it is crucial to develop methods that are specifically customized for the prediction of lncRNA–miRNA interactions in animals. Furthermore, current methods suffer from certain limitations, such as underutilization of node neighbourhood information and limited feature extraction capabilities.

To address these limitations and further enhance prediction performance, we propose a meta‐path‐based GAT model. This model integrates multiple matrices, including feature matrices derived from the doc2vec model, meta‐path‐based similarity matrices, and Gaussian kernel‐based similarity matrices. By incorporating these matrices into GAT, we can obtain a feature matrix that effectively captures node neighbourhood information. Ultimately, a scoring module is employed to predict lncRNA–miRNA interaction relationships. We aim to overcome the limitations of previous methods and achieve more accurate and comprehensive predictions.

## 2 MATERIALS AND METHODS

### 2.1 Data preparation

We utilize the research data provided by Wang et al. to conduct experimental predictions of lncRNA–miRNA interactions in animals,^34^ In our work, we refer to this dataset as the benchmark dataset. According to the data retrieved from LncACTdb 3.0 (http://bio‐bigdata.hrbmu.edu.cn/LncACTdb),^35^ a total of 1057 interactions between lncRNAs and miRNAs are identified, involving 284 unique lncRNAs and 520 miRNAs. The sequences of the lncRNAs are obtained from LNCipedia (https://lncipedia.org/)^36^ and NONCODE (http://www.noncode.org/),^37^ while the sequences of the miRNAs are obtained from miRBase (https://mirbase.org/).^38^

LncACTdb is a dedicated database that aims to gather, store and analyse experimentally verified gene interactions, while offering comprehensive annotations. The latest version, LncACTdb 3.0, features over 5000 experimentally confirmed gene interactions across 25 species and 537 diseases or phenotypes. Moreover, the database houses a collection of over 10,000 experimentally validated lncRNA biomarkers associated with tumour metastasis. Detailed information on the subcellular and extracellular localization of these biomarkers is manually curated from relevant literature and data sources. The database also offers comprehensive expression profiles of lncRNAs, mRNAs and miRNAs, along with clinical information about patients, such as metastasis, recurrence, drug resistance and prognosis. It is worth noting that all lncRNA–miRNA interactions recorded in the database have been experimentally validated. LNCipedia serves as a publicly accessible database for lncRNA sequences and annotations. It encompasses a vast collection of 127,802 transcripts and 56,946 genes. The database provides highly accurate and comprehensive annotations for lncRNAs, including transcripts from diverse sources, and offers functional annotations at both the gene and transcript levels. NONCODE is a comprehensive database specifically designed for collecting and annotating non‐coding RNAs, with a particular focus on lncRNAs in animals. The miRBase database serves as a searchable repository for published miRNA sequences and corresponding annotations.

### 2.2 MPGK‐LMI

**FIGURE 1:**
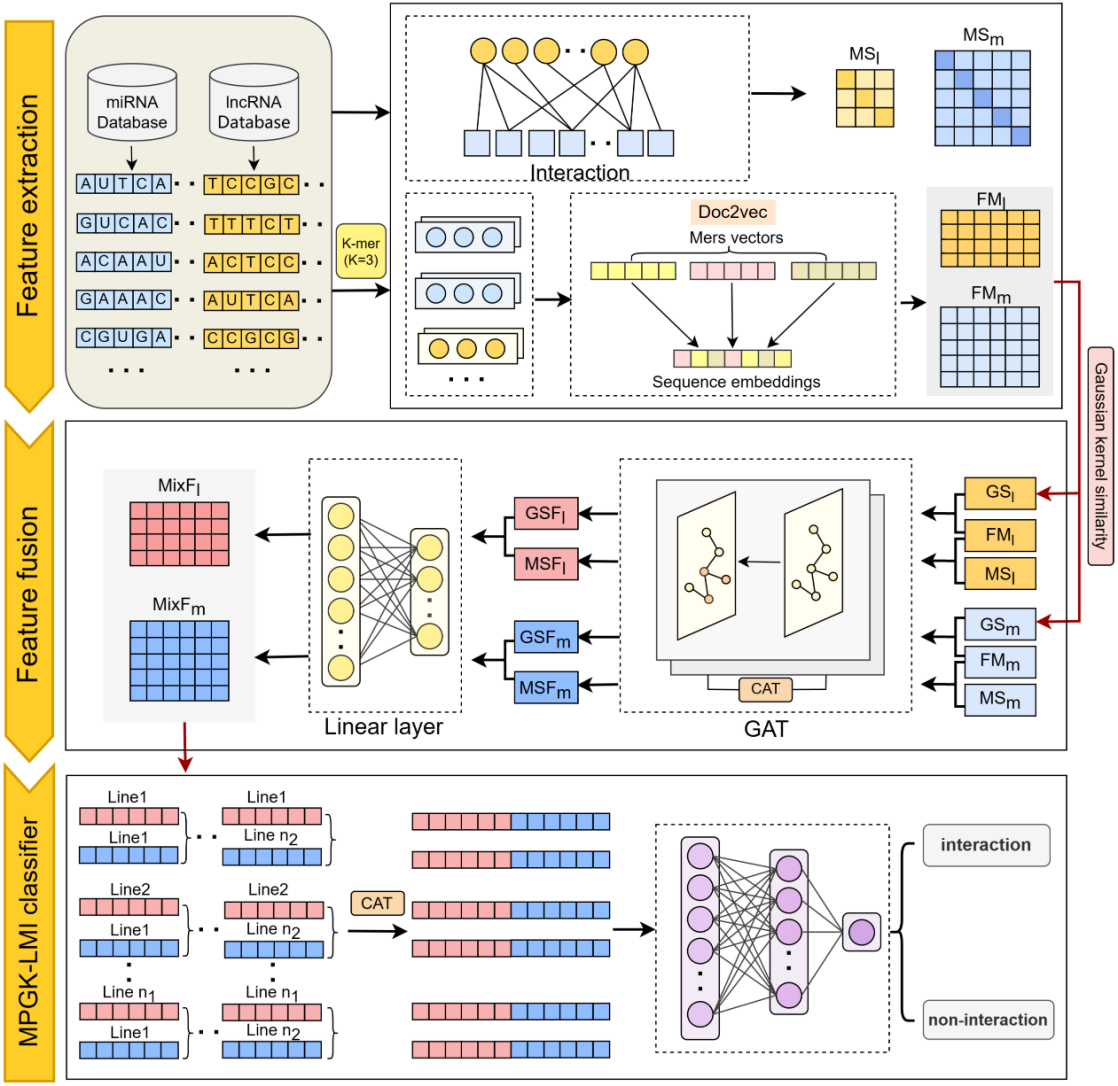
The workflow of MPG K‐LMI.

MPGK‐LMI consists of three modules: feature extraction, feature fusion and MPGK‐LMI classifier, which have a pivotal role in predicting lncRNA–miRNA interactions. Feature extraction module utilizes doc2vec to train on lncRNA and miRNA sequences, resulting in lncRNA feature matrix (FM_l_) and miRNA feature matrix (FM_m_). Subsequently, we calculate Gaussian kernel similarity based on FM_l_ and FM_m_, generating lncRNA Gaussian kernel similarity adjacency matrix (GS_l_) and miRNA Gaussian kernel similarity adjacency matrix (GS_m_). On the other hand, lncRNA and miRNA sequences can be transformed into lncRNA meta‐path similarity adjacency matrix (MS_l_) and miRNA meta‐path similarity adjacency matrix (MS_m_) through meta‐path similarity calculation. Feature fusion module aims to capture effective low‐dimensional representations from high‐dimensional features. It transforms and merges the original feature data to obtain feature matrices that incorporate neighbourhood information. This enables us to extract more meaningful and expressive feature representations from the complex high‐dimensional feature space. We utilize these six matrices obtained from the feature extraction module as input and train them using GAT, resulting in four matrices: the lncRNA feature matrix that combines Gaussian kernel similarity information (GSF_l_), the lncRNA feature matrix that integrates meta‐path similarity information (MSF_l_), the miRNA feature matrix that merges meta‐path similarity information (MSF_m_), and the miRNA feature matrix that encompasses Gaussian kernel similarity information (GSF_m_). Afterward, through concatenation and linear layers, we obtain the feature matrices that aggregate both types of neighbourhood information: MixF_l_ and MixF_m_. MPGK‐LMI classifier module is responsible for classifying lncRNA–miRNA interactions. The overall architecture of MPGK‐LMI is illustrated in Figure 1.

### 2.3 Feature extraction

#### 2.3.1 Feature matrix

We utilize doc2vec for feature extraction,^39^ which involves the following steps. First, we extract the required data from the database. For lncRNA and miRNA sequences, we divide them into overlapping subsequences of length *k*, also referred to as k‐mers.^40^ In our study, we found that a *k*‐value of 3 yields the best results, so we set the default k‐value to 3. This process allows us to break down the RNA sequences into multiple fixed‐length fragments, enabling more efficient handling of the input data. Next, we employ k‐mer counting techniques to process all lncRNA and miRNA sequences and record the frequency of occurrence for each k‐mer in these sequences. This transformation enables us to represent the RNA sequences based on the frequency of k‐mers. Subsequently, we utilize doc2vec to pretrain the RNA sequences and convert sequences of variable length into fixed‐length vectors. During the pretraining process, we employ an average operation on the sequence vectors to predict the next k‐mer segment within the sequence. This training procedure facilitates the capture of both local and global features of the RNA sequences and encodes them into fixed‐length embedding vectors. The trained doc2vec is then capable of generating embedding vectors for each RNA sequence. For this task, we employ distributed memory (DM) approach to train doc2vec. The essence of DM involves averaging the sequence vectors and k‐mer RNA segment vectors. This process enables the extraction of features that can predict the characteristics of the next k‐mer segment in the input sequence. In a sense, this can be seen as an extension of the continuous bag‐of‐words model in word2Vec. By utilizing this approach, we can capture both local and global features of the RNA sequence. The embedding of an RNA sequence using DM can be represented by the following formula:

(1) $$y_{t}=\text{soft}\max\left(b+\sum_{i=0}^{c}U_{i}W_{t-i}+\mathrm{VD}\right)$$

In this intricate process, the utilization of the output vector $y_{t}$, in conjunction with the context segments and sequence vector, facilitates the prediction of the subsequent k‐mer segment. To ensure proper normalization of the input, the softmax(·) function is applied, taking into account the bias term *b*. The vector representation $U_{i}$ precisely corresponds to the i‐th k‐mer segment within the processed RNA sequence.

Each weight, $W_{t-i}$, carries valuable information about the significance of the corresponding context k‐mer segment. The comprehensive weights *V* collectively reflect the importance and relevance of the entire RNA sequence, while the feature vector *D* encapsulates the distinctive characteristics of the processed RNA sequence. These carefully crafted vectors collectively capture the intrinsic features and semantic intricacies embedded within the RNA sequence. It enables the seamless transformation of RNA sequences with variable lengths into structured fixed‐length vector representations. By employing this sophisticated feature extraction technique, we not only gain a deeper understanding of the information encoded within RNA sequences but also unlock enhanced utilization of this valuable knowledge. Consequently, we can extract critical sequence features that are specific to lncRNAs and miRNAs, thereby paving the way for diverse applications across various research domains.

### 2.4 Meta‐path similarity matrix

A bipartite graph, also known as a bipartite network, is a special type of graph model in graph theory.^41^ Let *G* be a given undirected graph, and *V* be the set of vertices in the graph. We can partition *V* into two disjoint subsets, denoted as *A* and *B*. In a bipartite graph, *A* and *B* represent two separate sets of vertices, and each edge (*i*, *j*) in the graph connects two vertices, *i* and *j*, belonging to these two different vertex sets.

For the lncRNA–miRNA interaction data, we can represent it as a bipartite graph. In this case, lncRNA and miRNA belong to two disjoint sets of vertices, *A* and *B*, respectively. The interaction relationships are then represented by the edges connecting vertices from the different sets. By leveraging the properties of bipartite graphs, we can transform the interaction relationships in the benchmark dataset into meta‐paths, which allow us to generate two subgraphs. Meta‐paths serve as a way to represent patterns of relationships between nodes in a graph.^42^ By selecting different meta‐paths, we can capture various types of association patterns. Based on the structural information of lncRNA–miRNA interactions, we utilize meta‐paths as auxiliary tools to guide the process of similarity calculation, thereby generating MS_l_ for lncRNA. MS_l_ is a 284 × 284 matrix where each row and column represent different lncRNAs. Each element (*L*_*M*_, *L*_*N*_) in the matrix indicates whether there is an interaction between the lncRNA represented by the *M*th row and the lncRNA represented by the *N*th column. The element value corresponding to the interaction between two lncRNAs is 1 if present, and 0 otherwise. The calculation formula of MS_l_ is as follows:

(2) $$\text{Link}=\left(L_{M},L_{N}\right)=\text{Dbase}\left(M_{i},L_{M}\right)\wedge \text{Dbase}\left(M_{i},L_{N}\right)$$

here, *L* represents lncRNA, and *M* represents miRNA. *Dbase* represents the known interactions between lncRNA and miRNA. If an interaction exists, the value is 1; if not, it is 0. *M* and *N* belong to the set of lncRNAs, and ∀i∈miRNA. Similarly, the calculation method for MS_m_ for miRNA is similar to MS_l_. MS_m_ is a 520 × 520 matrix, where each element (*M*_*X*_, *M*_*Y*_) is calculated using the following formula:

(3) $$\text{Link}=\left(M_{X},M_{Y}\right)=\text{Dbase}\left(L_{j},M_{X}\right)\wedge \text{Dbase}\left(L_{j},M_{Y}\right)$$

here, *X* and *Y* belong to the set of miRNAs, and ∀j∈lncRNA.

Subsequently, we use the calculated meta‐path similarity information matrices to update the feature matrices for lncRNA and miRNA.

### 2.5 Gaussian kernel similarity matrix

In the context of known lncRNA–miRNA interaction relationships, our objective is to consider the information between nodes and compute the similarity between lncRNA–lncRNA and miRNA–miRNA pairs. For a given pair of lncRNA nodes, $l_{i}$ and $l_{j}$, we can compute their similarity using the Gaussian kernel function, denoted as ${\mathrm{GS}}_{l}\left(i,j\right)$. The calculation formula is as follows:

(4) $${\mathrm{GS}}_{l}\left(i,j\right)=\exp\left(\frac{-{\left\|l_{i}-l_{j}\right\|}^{2}}{2*\sigma^{2}}\right)$$

here, $l_{i}$ and $l_{j}$ represent the lncRNA nodes. The Euclidean distance between the feature vectors of $l_{i}$ and $l_{j}$ is denoted as $\left\|l_{i}-l_{j}\right\|$, and σ is the bandwidth parameter of the Gaussian kernel function, and it is set to 1.

Similarly, for a given pair of miRNA nodes, $m_{i}$ and $m_{j}$, we can compute their similarity using the Gaussian kernel function, denoted as ${\mathrm{GS}}_{m}\left(i,j\right)$. The calculation formula is as follows:

*GS*_*m*_

(5) $${\mathrm{GS}}_{m}\left(i,j\right)=\exp\left(\frac{-{\left\|m_{i}-m_{j}\right\|}^{2}}{2*\sigma^{2}}\right)$$

here, $m_{i}$ and $m_{j}$ represent the miRNA nodes. The Euclidean distance between the feature vectors of $m_{i}$ and $m_{j}$ is denoted as $\left\|m_{i}-m_{j}\right\|$, and σ is the bandwidth parameter of the Gaussian kernel function, and it is set to 1.

These similarity measures provide information about the relationships and similarities among different lncRNAs and miRNAs in the network. The computation of Gaussian kernel similarity allows us to consider both the features of the nodes and the degree of dissimilarity between them. When the difference between two nodes is small, the Gaussian kernel similarity approaches 1, indicating a higher similarity in the feature space. Conversely, when the difference between nodes is large, the Gaussian kernel similarity approaches 0, indicating a lower similarity in the feature space. By calculating GS_l_ and GS_m_, we can obtain two similar matrices composed of node elements. These matrices provide valuable information about the similarity patterns and associations among lncRNA–lncRNA pairs and miRNA–miRNA pairs. Subsequently, we use GS_l_ and GS_m_ to respectively update FM_l_ and FM_m_.

### 2.6 Feature fusion

GAT is a widely used model in the field of graph neural networks. It possesses strong expressive capabilities when dealing with graph‐structured data and enables flexible modelling of relationships between different nodes. The core principle of GAT lies in its utilization of self‐attention mechanisms to aggregate information from nodes within the graph. By computing attention weights between nodes, GAT can dynamically allocate importance to different node pairs. This adaptability allows GAT to learn diverse relationships across varying node connection patterns and graph topologies.

In MPGK‐LMI, we employ GAT to obtain GSF_l_, MSF_l_, MSF_m_, and GSF_m_.^43^ Our GAT architecture consists of two attention layers. Within each attention layer, we employ the adjacency matrix to compute relational weights between nodes, which are then applied to the feature matrix. This approach enables us to consider both the inherent features of nodes themselves and the relationships between nodes and their neighbouring counterparts. Our attention computation process involves performing row‐wise attention calculations using GS_l_ and FM_l_, MS_l_ and FM_l_, GS_m_ and FM_m_, and MS_m_ and FM_m_. During each attention computation step, we leverage an attention mechanism to allocate weights to distinct pairs of nodes. These weights denote the significance and correlation among nodes. In the case of GAT, for a given node *i*, we utilize the following formula to compute the attention weights between node *i* and its neighbouring node *j*:

(6) eij=LeakyReLUa⊤Wxi∥Wxj

where $\left\lfloor \left\lceil {\mathrm{Wx}}_{i}^{k}∥{\mathrm{Wx}}_{j}^{k}\right\rceil \right\rfloor$ is the concatenation of the feature vectors of node *i* and node *j*, and || denotes the concatenation operation. LeakyReLU is a leaky rectified linear unit activation function is applied to introduce non‐linearity. Next, the attention weights are normalized using the softmax function to obtain attention coefficients $a_{\mathrm{ij}}$:

(7) $$a_{\mathrm{ij}}=\frac{\exp\left(e_{\mathrm{ij}}\right)}{\sum_{k\in N_{i}}\exp\left(e_{\mathrm{ik}}\right)}$$

here, $N_{i}$ refers to the set of adjacent nodes for node *i*. Then, the features of the neighbouring nodes for *i* are aggregated using the attention coefficients to obtain the aggregated feature representation of *i*. Next, the $a_{\mathrm{ij}}$ are used to compute a weighted sum of the feature representations of neighbouring nodes for each node *i*:

(8) $$h_{i}=\sigma \left(\sum_{j\in N_{i}}a_{\mathrm{ij}}\left({\mathrm{Wx}}_{j}\right)\right)$$

In MPGK‐LMI, we include four identical GATs. Each GAT takes GS_l_ and FM_l_, MS_l_ and FM_l_, MS_m_ and FM_m_, and GS_m_ and FM_m_ as inputs, respectively, to acquire feature matrices that aggregate neighbourhood information. It is noteworthy that, to facilitate information propagation across different layers and capture additional graph information, we concatenate the initial feature matrix with the feature matrix obtained after two layers of attention computations. Then, we apply a linear layer to decrease the matrix's dimensionality and obtain lower‐dimensional representation, MixF_l_ and MixF_m_.

### 2.7 MPGK‐LMI classifier

As shown in Figure 1, MPGK‐LMI concatenates the features from MixF_l_ and MixF_m_ matrices when predicting lncRNA–miRNA interactions. Specifically, the rows from line 1 to line n_1_ in MixF_l_ matrix are concatenated with the rows from line 1 to line n_2_ in MixF_m_ matrix. This selective concatenation of the corresponding rows from the two input matrices, MixF_l_ and MixF_m_, produces a new feature vector. This concatenated feature vector is then fed into the scoring module. During the training process, we use the binary cross‐entropy loss function. The binary cross‐entropy loss function is a widely employed loss function in binary classification tasks. It quantifies the discrepancy between the predicted outcomes of MPGK‐LMI and the actual labels in a binary classification problem. The binary cross‐entropy loss function can be expressed as:

(9) $$L=-\left(y^{*}\log\left(p\right)+\left(1-y\right)*\log\left(1-p\right)\right)$$

here, *L* represents the loss value, *y* is the true label (0 or 1), and *p* is the predicted probability by MPGK‐LMI. When y=1, the first term of the loss function, $-y^{*}\log\left(p\right)$, contributes more and penalizes MPGK‐LMI for inaccurate predictions of positive samples. When y=0, the second term of the loss function, $-\left(1-y\right)*\log\left(1-p\right)$, contributes more and penalizes MPGK‐LMI for inaccurate predictions of negative samples. By minimizing the binary cross‐entropy loss function, we can train MPGKLMI to make more accurate predictions for binary classification problems.

## 3 RESULTS

### 3.1 Performance evaluation

To address the issue of data imbalance, we partition the interacting samples from the benchmark dataset during the training process and validation sets, with a ratio of 8:2. Next, to create a balanced training set, we ensure an equal number of non‐interacting samples are selected to match the number of interacting samples. This ensures equal representation of interacting and non‐interacting samples.

To evaluate the performance of MPGK‐LMI, we utilize commonly employed binary classification metrics, including area under the ROC curve (AUC), area under the precision‐recall curve (AUPR), accuracy (ACC), F1‐score, and precision.

AUC stands as a pivotal metric employed for assessing model efficacy. It measures the ability of model to correctly classify positive and negative samples at different classification thresholds. A greater AUC value signifies superior model performance. The calculation of AUC involves comparing true positive rate (TPR) and false positive rate (FPR). TPR calculates the proportion of correctly predicted positive samples among all existing lncRNA–miRNA interactions. FPR measures the ratio of falsely predicted positive samples among all non‐existent lncRNA–miRNA interactions. TPR and FPR can be calculated using the following formulas:

(10) $$\mathrm{TPR}=\frac{\mathrm{TP}}{\mathrm{TP}+\mathrm{FN}}$$

(11) $$\mathrm{FPR}=\frac{\mathrm{FP}}{\mathrm{TN}+\mathrm{FP}}$$

here, TP represents the count of properly forecasted existing lncRNA–miRNA interactions, TN represents the count of correctly predicted non‐existing lncRNA–miRNA interactions, FP represents the count of incorrectly predicted existing lncRNA–miRNA interactions, and FN represents the count of incorrectly predicted non‐existing lncRNA–miRNA interactions.

AUPR is another important evaluation metric that is particularly advantageous when dealing with imbalanced classes or skewed datasets. This metric assesses the accuracy of a model in predicting lncRNA–miRNA interactions by approximating AUPR using the trapezoidal rule. The relevant formula for calculation is as follows:

(12) $$\text{Recall}=\frac{\mathrm{TP}}{\mathrm{TP}+\mathrm{FP}}$$

(13) $$\text{Precision}=\frac{\mathrm{TP}}{\mathrm{TP}+\mathrm{FP}}$$

(14) $$\text{AUPR}=\sum_{i=1}^{n}\left(\text{Recall}\left[i\right]-\text{Recall}\left[i-1\right]\right)*\text{Precision}\left[i\right]$$

here, Recall is an important metric in the evaluation of binary classification models. It quantifies the ratio of accurately predicted lncRNA–miRNA interactions among all genuine positive instances. Precision is one of the metrics used to assess the accuracy of positive predictions made by the model. It represents the accuracy of the model in correctly predicting the existence of lncRNA–miRNA interactions. A high Precision indicates that the model can accurately identify true positive samples when predicting lncRNA–miRNA interactions. In equation 14, *n* represents the number of different thresholds. $\text{Recall}\left[i\right]$ and $\text{Precision}\left[i\right]$ respectively denote the Recall and Precision at the i‐th threshold.

ACC is one of the metrics used to evaluate the accuracy of model predictions. It represents the proportion of correctly predicted interactions out of the total number of interactions. A high ACC indicates that the model has a higher level of ACC in predicting lncRNA–miRNA interactions. The formula for calculating ACC is as follows:

(15) $$\mathrm{ACC}=\frac{\mathrm{TP}+\mathrm{FN}}{\mathrm{TP}+\mathrm{TN}+\mathrm{FP}+\mathrm{FN}}$$

F1‐score is one of the metrics that comprehensively evaluates the performance of a classification model. It takes into account both Precision and Recall and provides a balanced measure of the model's performance. It is based on the correct predictions of lncRNA–miRNA interactions, incorrect predictions of non‐interactions, and correct predictions of non‐interactions. A higher F1‐score indicates the better overall performance of the model in predicting lncRNA–miRNA interactions. The formula for calculating F1‐score is as follows:

(16) $$F1‐\text{score}=\frac{2^{*}{\text{Precision}}^{*}\text{Recall}}{\text{Precision}+\text{Recall}}$$

### 3.2 Comparison with other methods

To assess the performance of MPGK‐LMI, we conduct a comparison with three widely‐used prediction models, namely preMLI,^44^ SPGNN,^34^ and LPICGAE.^45^

1. PreMLI is a deep learning model that utilizes rna2vec pre‐training and deep feature mining mechanisms for predicting lncRNA–miRNA interactions.
2. SPGNN is a sequence pre‐trained graph neural network that combines pre‐training and fine‐tuning stages to predict lncRNA–miRNA interactions.
3. LPICGAE introduces a novel deep‐learning approach that combines graph autoencoders to predict potential interactions between human lncRNAs and proteins.

**FIGURE 2:**
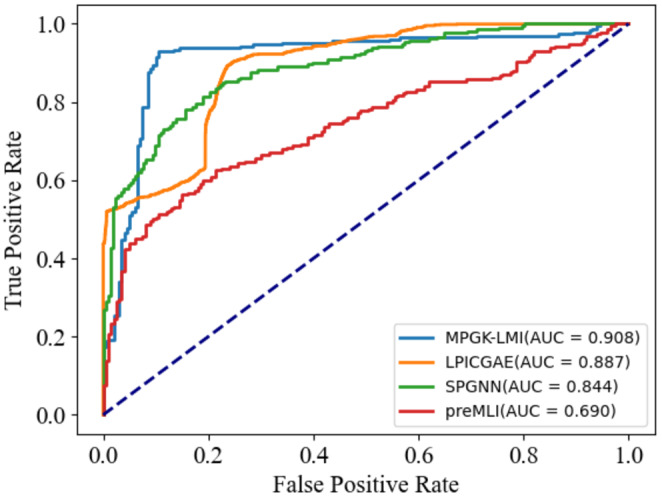
ROC curves of MPGK‐LMI and comparison methods under benchmark dataset.

Table 1 shows that MPGK‐LMI has AUC of 0.9077, which is 21.74%, 6.32% and 2.07% higher than that of preMLI, SPGNN, and LPICGAE, respectively. This indicates that MPGK‐LMI outperforms the other three models in overall performance and demonstrates excellent classification ability. Figure 2 displays the ROC curve. MPGK‐LMI has AUPR of 0.9327, which is 23.42%, 8.18% and 44.49% higher than that of preMLI, SPGNN and LPICGAE, respectively. This suggests that MPGK‐LMI achieves higher Precision at various Recall levels and exhibits excellent predictive performance. MPGK‐LMI records ACC of 0.9080, which surpasses that of preMLI and SPGNN by 22.04% and 2.87% respectively while falling 8.38% lower than that of LPICGAE. This indicates that MPGK‐LMI performs better in terms of ACC compared to preMLI and SPGNN, but slightly lower than LPICGAE. Furthermore, MPGK‐LMI achieves F1‐score of 0.9143, which is 25.71%, 15.39% and 47.86% higher than that of preMLI, SPGNN, and LPICGAE, respectively. This indicates that MPGK‐LMI performs well in overall performance and achieves a good balance between Precision and Recall. It is worth mentioning that the Precision metric of MPGK‐LMI is 0.8739, while LPICGAE has a Precision of 0.2100. This emphasizes the exceptional correctness of MPGK‐LMI in predicting positive samples and its capability to accurately identify positive instances.

**TABLE 1:** Comparative analysis of MPGK‐LMI and other three methods under benchmark dataset.

Method	AUC	AUPR	ACC	F1‐score	Precision
MPGK‐LMI	**0.9077**	**0.9327**	0.9080	**0.9143**	**0.8739**
preMLI	0.6903	0.6985	0.6876	0.6572	0.7326
SPGNN	0.8445	0.8509	0.8802	0.7604	0.7914
LPICGAE	0.8870	0.4878	**0.9918**	0.4357	0.2100

In summary, according to Table 1, MPGK‐LMI demonstrates superior performance in terms of overall performance, classification ability, and predictive performance compared to the other three models.

PreMLI exclusively relies on the rna2vec algorithm for pretraining, which provides word vector representations of RNA sequences. Similarly, SPGNN relies solely on the doc2vec algorithm for pretraining to obtain word vector representations. However, it is important to note that these representations may not capture the intricate structural and functional information inherent in RNA sequences. In contrast, MPGK‐LMI takes a more sophisticated approach by leveraging both Gaussian kernel similarity and meta‐path similarity information. This allows MPGK‐LMI to learn more meaningful and informative feature representations of RNA sequences. By incorporating these additional similarity measures, MPGK‐LMI can better capture the complex relationships and similarities between RNA molecules. As a result of its more advanced methodology, MPGK‐LMI demonstrates superior performance compared to preMLI and SPGNN across multiple evaluation metrics, including AUC, AUPR, ACC, F1‐score and precision. These findings highlight the effectiveness of MPGK‐LMI in accurately capturing the essential characteristics of RNA sequences. LPICGAE leverages a variational graph autoencoder to acquire low‐dimensional representations of both lncRNA and miRNA. Additionally, it utilizes a graph autoencoder to reconstruct the adjacency matrix and infer plausible interactions. However, the performance of LPICGAE may be limited by its feature representation capability. Unfortunately, LPICGAE may struggle to fully grasp the key information between lncRNA and miRNA due to its limited feature representation capacity. Consequently, this limitation can negatively impact the exactness of interaction predictions. In contrast, MPGK‐LMI improves predictive exactness by incorporating Gaussian kernel similarity. This enhancement enables a better capture of important information between lncRNA and miRNA. By leveraging Gaussian kernel similarity, MPGK‐LMI excels in capturing complex relationships and crucial features between lncRNA and miRNA. As a result, it demonstrates superior performance in evaluation metrics such as AUC, AUPR, F1‐score and precision. However, it is worth noting that the ACC is slightly lower compared to LPICGAE. This is because LPICGAE tends to overfit when dealing with the benchmark dataset, as it does not specifically address the issue of data imbalance. In contrast, MPGK‐LMI focuses more on addressing the problem of imbalanced data, thereby avoiding overfitting.

### 3.3 Ablation experiments

MPGK‐LMI comprises two crucial feature matrices, one incorporating aggregated meta‐path similarity information and the other incorporating aggregated Gaussian kernel similarity information. In this section, we perform ablation experiments to evaluate how each component influences the predictive performance of MPGK‐LMI on the benchmark dataset.

1. MPGK‐LMI‐OG: We exclude the feature matrix incorporating aggregated meta‐path similarity information from MPGK‐LMI. That is, in the feature fusion step, we only input GS_l_, GS_m_, FM_l_ and FM_m_ into GAT for training. We then feed the obtained results into the classifier for prediction.
2. MPGK‐LMI‐OM: We exclude the feature matrix incorporating aggregated Gaussian kernel similarity information from MPGK‐LMI. That is, in the feature fusion step, we only input MS_l_, MS_m_, FM_l_ and FM_m_ into GAT for training. We then feed the obtained results into the classifier for prediction.

**FIGURE 3:**
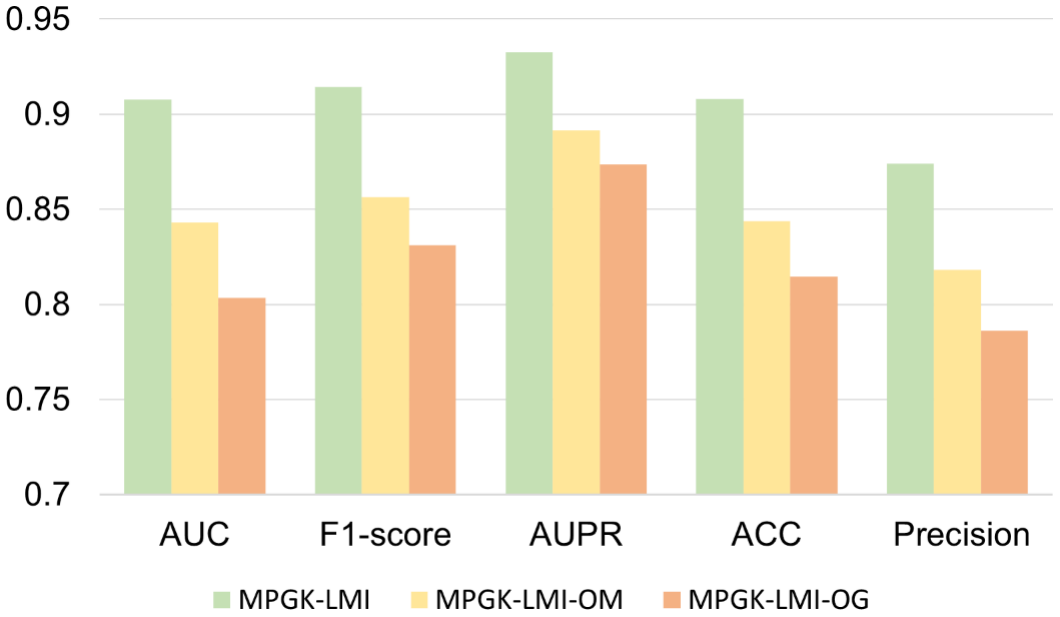
Comparison analysis between MPGK‐LMI and its ablation experiments.

Figure 3 illustrates the AUC performance comparison of MPGK‐LMI and its two variants on the benchmark dataset. As shown in Table 2, MPGK‐LMI outperforms MPGK‐LMI‐OG and MPGK‐LMI‐OM in terms of performance. MPGK‐LMI achieves AUC of 0.9077, which is 10.43% and 6.48% higher than that of MPGK‐LMI‐OM and MPGK‐LMI‐OM, respectively. Moreover, MPGK‐LMI exhibits higher F1‐score than that of MPGK‐LMI‐OG and MPGK‐LMI‐OM by 8.32% and 5.79%, respectively. Additionally, MPGK‐LMI achieves higher AUPR than that of MPGK‐LMI‐OM and MPGK‐LMI‐OM by 5.91% and 4.13%, respectively. Furthermore, MPGK‐LMI demonstrates higher ACC than that of MPGK‐LMI‐OG and MPGK‐LMI‐OM by 9.33% and 6.42%, respectively. The meta‐path similarity approach captures more complex associations and pathway patterns by considering the intermediate nodes between lncRNA, miRNA, and their interactions. However, it does not account for the dynamics and temporal information of the interactions. On the other hand, Gaussian kernel similarity is a simple and intuitive similarity measure that enables a quick assessment of the interaction likelihood based on the similarity between feature vectors of lncRNA and miRNA. However, Gaussian kernel similarity may overlook the contextual information of lncRNA and miRNA in biological contexts. By combining the Gaussian kernel similarity matrix and the meta‐path similarity matrix, a fused feature matrix is created, providing more accurate feature information and improving predictive accuracy. This integration allows MPGK‐LMI to achieve superior performance compared to MPGK‐LMI‐OG and MPGK‐LMI‐OM in all performance metrics.

**TABLE 2:** Comparison analysis between MPGK‐LMI and its ablation experiments on benchmark dataset.

Method	AUC	AUPR	ACC	F1‐score	Precision
MPGK‐LMI	**0.9077**	**0.9327**	**0.9080**	**0.9143**	**0.8739**
MPGK‐LMI‐OM	0.8429	0.8914	0.8438	0.8564	0.8180
MPGK‐LMI‐OG	0.8034	0.8736	0.8147	0.8311	0.7861

### 3.4 Analysis of parameters

In this section, we examine how three critical parameters affect the performance of MPGK‐LMI: the number of graph attention layers (GAL), different learning rates (LR), and varying numbers of hidden layer nodes (HLN).

The selection of GAL is a nuanced consideration without a one‐size‐fits‐all solution. It depends on various factors, including the specific dataset and task at hand, each of which may impose different requirements on the layer count. Increasing GAL can boost MPGK‐LMI's complexity and expressive capacity, enabling it to capture intricate relationships and patterns within graph data more effectively. By gradually learning higher‐order node relationships, MPGK‐LMI enhances its representation capabilities. However, excessive layer usage can result in overfitting to the training data, impeding the generalization capability of MPGK‐LMI. Moreover, augmenting GAL escalates MPGK‐LMI's parameter count, computational complexity, and training difficulty. The scale and intricacy of the dataset also influence the optimal layer count. Smaller and simpler datasets often suffice with shallower models that can adequately fit the data. Conversely, more extensive and complex datasets may benefit from increased layer depth to enhance model performance. In our study, we conduct experiments employing one, two, and three GAL. As indicated in Table 3, compared to the cases where GAL is 1 and 3, the AUC increases by 5.73% and 1.59%, respectively, the AUPR increases by 3.91% and 1.07% respectively, the ACC increases by 5.42% and 1.41% respectively, the F1‐score increases by 4.62% and 1.19% respectively, and the Precision increases by 7.15% and 2.12%, respectively. Therefore, MPGK‐LMI achieves the best performance when employing two GAL.

**TABLE 3:** The performance of MPGK‐LMI under different GAL values.

GAL	AUC	AUPR	ACC	F1‐score	Precision
1	0.8504	0.8936	0.8538	0.8681	0.8024
2	**0.9077**	**0.9327**	**0.9080**	**0.9143**	**0.8739**
3	0.8918	0.9220	0.8939	0.9024	0.8527

The LR is a critical hyperparameter in deep learning models as it determines the step size of parameter updates during training.^46^ The choice of LR can have a significant impact on MPGK‐LMI training and performance. Setting LR too high can lead to large parameter updates, causing MPGK‐LMI to fail to converge or even diverge during training. This can result in unstable behaviour, with the loss function exhibiting significant fluctuations, and MPGK‐LMI being unable to reach an optimal solution. Conversely, setting LR too low can result in slow MPGK‐LMI convergence. In such cases, our model may require a larger number of iterations to achieve satisfactory performance. However, excessively small LR might cause MPGK‐LMI to get trapped in local optima or saddle points, preventing further optimization. Selecting appropriate LR involves careful tuning and experimentation, taking into account the specific tasks and characteristics of MPGK‐LMI. As evident from Table 4, when LR is set to 0.0001, the model achieves the best performance. Compared to the cases where LR is 0.001 and 0.00001, the AUC is 0.65% and 15.43% higher respectively, the AUPR is 0.01% and 9.77% higher respectively, the ACC is 0.71% and 16.04% higher respectively, and the F1‐score is 0.89% and 18.25% higher respectively. HLN is a crucial hyperparameter in deep learning models, as it determines the capacity and complexity of MPGK‐LMI's hidden representations.^47^ It has implications for MPGK‐LMI's expressive power, training speed, and generalization performance. Augmenting HLN can improve MPGK‐LMI's capacity to capture intricate patterns and relationships within the data. The availability of more HLN provides MPGK‐LMI with increased flexibility to learn intricate nonlinear mappings. However, configuring HLN to excessively high values could result in overfitting, causing MPGK‐LMI to become overly tailored to the training data and resulting in poor generalization to unseen examples.

**TABLE 4:** The performance of MPGK‐LMI under different LR values.

LR	AUC	AUPR	ACC	F1‐score	Precision
0.001	0.9012	0.9326	0.9009	0.9054	**0.8741**
0.0001	**0.9077**	**0.9327**	**0.9080**	**0.9143**	0.8739
0.00001	0.7534	0.8350	0.7476	0.7318	0.7277

Moreover, a large HLN can increase MPGK‐LMI's parameter count and computational complexity, resulting in longer training times and higher resource requirements. The selection of appropriate HLN requires a careful balance between MPGK‐LMI's capacity, training efficiency, and the characteristics of the dataset. There is no universal rule, and the optimal HLN should be determined through empirical experimentation and hyperparameter tuning. As evident from Table 5, the model achieves the best performance when HLN is set to 512. Compared to the best results obtained with HLN of 128, 256, and 1024, the AUC is 1.46% higher, the AUPR is 0.77% higher, the ACC and F1‐score are 1.41% higher, and the Precision is 1.39% higher.

**TABLE 5:** The performance of MPGK‐LMI under different HLN values.

HLN	AUC	AUPR	ACC	F1‐score	Precision
128	0.8829	0.9167	0.8844	0.8928	0.8445
256	0.8931	0.925	0.8939	0.9002	0.8600
512	**0.9077**	**0.9327**	**0.9080**	**0.9143**	**0.8739**
1024	0.8701	0.9071	0.8726	0.8836	0.8265

### 3.5 Case study

To further validate the predictive capacity of MPGK‐LMI in identifying potential interactions between lncRNAs and miRNAs, we conduct a case study focusing on two specific lncRNAs, namely MALAT1 and HOTAIR. In this study, we conduct model training and identify the most effective model generated by MPGK‐LMI, which possesses the ability to generate node features for each RNA. The ranking of potential interactions is determined based on the evaluation of relevance scores derived from these node features.

MALAT1, also known as NEAT2, is a lncRNA that was first reported in 2003. It spans a length of 8779 nucleotides and is located on chromosome 11q13.^48^ MALAT1 is among the most evolutionarily conserved lncRNAs in mammals and exhibits high abundance within the nucleus of normal tissue cells. The interactions between MALAT1 and numerous miRNAs hold significant biological significance. To explore the miRNAs that potentially interact with MALAT1, we conduct an analysis using the MPGK‐LMI algorithm. Notably, we focus on MALAT1's interactions and exclude any other interactions. The results of this analysis are presented in Table 6. Remarkably, out of the top 10 predicted miRNAs, 7 miRNAs have been supported by previous research evidence. One of the identified interactions involves MALAT1's ability to modulate the expression level of miR‐122 in hepatocytes. MiR‐122 is pivotal in hepatic regulation, contributing to the control of processes such as cholesterol metabolism, lipid homeostasis, and viral infection.^49^ Additionally, the upregulation of MALAT1 has been observed to suppress the expression of miR‐145‐5p, consequently impacting various biological processes, tumour cell proliferation, migration, invasion, as well as apoptosis and angiogenesis in cardiovascular diseases are among the processes influenced by miR‐122.^50^ Furthermore, upregulation of MALAT1 in human breast cancer cells has been identified to result in the downregulation of miR‐146b‐5p expression, thereby promoting cell proliferation and invasion.^51^ In embryonic stem cells, MALAT1 has been shown to modulate self‐renewal and differentiation capabilities through its interaction with miR‐509.^52^ Similarly, MALAT1 has been implicated in the regulation of multiple biological processes, including tumour cell proliferation, migration, and invasion, as well as apoptosis and angiogenesis in cardiovascular diseases, through its binding to miR‐145‐5p.^53^ Moreover, in gastric cancer, the upregulation of MALAT1 has been associated with the inhibition of miR‐370‐3p expression, thereby facilitating tumour cell proliferation and invasion.^54^ Additionally, studies have demonstrated that the increased expression of MALAT1 leads to the inhibition of miR‐218 expression, subsequently promoting cancer cell proliferation, invasion, and metastasis. These findings provide insight into the intricate regulatory roles of MALAT1 in various biological processes through its interactions with specific miRNAs. The elucidation of such interactions contributes to our understanding of the underlying mechanisms and potential therapeutic targets associated with lncRNA–miRNA interactions.

**TABLE 6:** The top 10 predicted results of lncRNA MALAT1 related miRNAs based on MPGK‐LMI.

Rank	MiRNA	PMID
1	miR‐186	Unknown
2	miR‐122	27486823
3	miR‐145‐5p	29633510
4	miR‐1180	Unknown
5	miR‐146b‐5p	33677577
6	miR‐93‐5p	Unknown
7	miR‐509	28560950
8	miR‐145‐5p	29633510
9	miR‐370‐3p	32998017
10	miR‐218	28069878

HOTAIR, also known as HOX transcript antisense RNA, is a lncRNA that has been extensively studied in the context of various miRNAs.^55^ In our analysis, we specifically focus on the interactions involving HOTAIR and exclude any other related interactions. By utilizing MPGK‐LMI, we predict the miRNAs that potentially interact with HOTAIR, and the results are presented in Table 7. Notably, 6 out of the top 10 predicted miRNAs have been supported by research evidence available in PubMed. In breast cancer, upregulation of HOTAIR has been shown to downregulate the expression of miR‐454‐3p, thereby promoting tumour cell proliferation and invasion.^56^ Similarly, in gastric cancer, upregulation of HOTAIR has been found to suppress the expression of miR‐130a, leading to enhanced cancer cell proliferation and metastasis.^57^ The interplay of HOTAIR and miR‐214‐3p has been implicated in the regulation of cancer cell proliferation and invasion. In specific contexts such as lung cancer and breast cancer, upregulation of HOTAIR has been observed to downregulate the expression of miR‐214‐3p.^58^ In liver cancer, increased expression of HOTAIR has been demonstrated to suppress the expression of miR‐17‐5p, resulting in increased liver cancer cell proliferation and metastasis.^59^ Additionally, HOTAIR has been found to interact with miR‐193a, influencing tumour cell growth and invasion. In breast cancer and colorectal cancer, the upregulation of HOTAIR has been associated with the downregulation of miR‐193a expression.^60^ Furthermore, upregulation of HOTAIR has been reported to suppress the expression of miR‐19, contributing to enhanced proliferation and invasion of breast cancer cells.^61^ These findings offer valuable insights into the regulatory mechanisms concerning HOTAIR and its interactions with specific miRNAs. Understanding these interactions contributes to our knowledge of the underlying molecular processes and potential therapeutic targets associated with lncRNA–miRNA interactions.

**TABLE 7:** The top 10 predicted results of lncRNA HOTAIR related miRNAs based on MPGK‐LMI.

Rank	MiRNA	PMID
1	miR‐454‐3p	28182000
2	miR‐130a	33398378
3	miR‐193a‐3p	Unknown
4	miR‐508	Unknown
5	miR‐34b‐3p	Unknown
6	miR‐133a‐3p	Unknown
7	miR‐214‐3p	34320988
8	miR‐17‐5p	34180119
9	miR‐193a	25979172
10	miR‐19	28316060

Through case studies of two key lncRNAs, MALAT1 and HOTAIR, we further validate the effectiveness of MPGK‐LMI in predicting potential lncRNA–miRNA interactions. The analysis results show that the model generated by MPGK‐LMI is able to accurately predict multiple lncRNA–miRNA interactions that have been experimentally verified, providing important evidence for further exploring the regulatory roles of these lncRNAs in biological processes. These findings not only deepen our understanding of the molecular mechanisms of key lncRNAs such as MALAT1 and HOTAIR and their interactions with specific miRNAs, but also lay the foundation for potential diagnostic and therapeutic applications utilizing these interactions. Overall, this case study further demonstrates the effectiveness and application prospects of MPGK‐LMI in predicting lncRNA–miRNA interaction networks.

## 4 DISCUSSION AND CONCLUSION

In recent years, it has been discovered that lncRNA–miRNA interactions play a critical role in biological processes. However, determining these interaction relationships remains a time‐consuming and labour‐intensive task. To address this issue, we propose a method named MPGK‐LMI based on GAT to predict the interactions between lncRNA and miRNA. Our approach utilizes a meta‐path similarity matrix, Gaussian kernel similarity matrix, and feature matrix to construct a graph structure, and incorporates attention mechanisms to capture the relationships and importance between nodes. Through multiple layers of attention computations, we obtain more comprehensive and accurate feature representations. Subsequently, we decode and score the final embeddings to obtain prediction results. We compare MPGK‐LMI with previous approaches using various evaluation metrics such as AUC, AUPR, ACC, F1‐score and Precision on the benchmark dataset. Experimental results demonstrate that our method performs favourably in predicting lncRNA–miRNA interactions. We also provide detailed explanations of the feature matrix, feature fusion and MPGK‐LMI classifier modules, as well as present experimental results and analysis. Our method effectively utilizes the graph structure data to incorporate relationship and contextual information, providing a foundation for further research and application of lncRNA–miRNA interactions.

MPGK‐LMI incorporates two different types of feature information, Gaussian kernel similarity, and meta‐path similarity, and aggregates them through the feature fusion module. This approach allows us to leverage the strengths of both features, leading to more comprehensive and accurate feature representations, thus improving predictive performance. Feature fusion module can extract effective low‐dimensional representations from high‐dimensional feature space. By aggregating neighbourhood information and using multiple linear layers, this module captures complex correlations and pathway patterns, generating feature matrices that incorporate contextual information. In the graph attention part, we connect the original neighbourhood information with the attention‐computed feature matrices to facilitate the flow of information between different layers. This enables richer and more accurate corrections to the feature information at lower layers, thereby enhancing the quality and expressive power of the feature representation.

However, MPGK‐LMI still has certain limitations. First, we have a limited amount of validation data, with a smaller number of known lncRNA–miRNA interaction samples compared to unknown samples. Second, factor affecting the performance of MPGK‐LMI is the similarity between lncRNA and miRNA, and integrating more biological information could enhance the reliability of similarity measures. With an increase in the number of experimental validation samples and improvements in parameter optimization algorithms, we anticipate that the predictive performance of MPGK‐LMI will continue to enhance. Furthermore, as deep learning research progresses and data volume increases, we anticipate that deep learning will replace traditional machine learning methods in predicting lncRNA–miRNA interactions, particularly with the emergence of graph‐based deep learning models such as GCN networks, which are well‐suited for handling this type of data. We look forward to the future application of related prediction models in the field of bioinformatics research.

## AUTHOR CONTRIBUTIONS

**Jingxuan Xie:** Data curation (equal); investigation (equal); methodology (equal); writing – original draft (equal). **Peng Xu:** Data curation (equal); investigation (equal); validation (equal); writing – original draft (equal). **Ye Lin:** Data curation (equal); investigation (equal); validation (equal). **Manyu Zheng:** Data curation (equal); validation (equal); visualization (equal). **Jixuan Jia:** Investigation (equal); validation (equal); visualization (equal). **Xinru Tan:** Investigation (equal); validation (equal); visualization (equal). **Jianqiang Sun:** Conceptualization (equal); methodology (equal); project administration (equal); writing – review and editing (equal). **Qi Zhao:** Conceptualization (equal); funding acquisition (equal); methodology (equal); project administration (equal); resources (equal); writing – review and editing (equal).

## FUNDING INFORMATION

This study is supported by Natural Science Foundation of Liaoning Province (Grant No. 2023‐MS‐288), Fundamental Research Funds for the Liaoning Universities.

## CONFLICT OF INTEREST STATEMENT

The authors declare that the research was conducted in the absence of any commercial or financial relationships that could be construed as a potential conflict of interest.

## Data Availability

The source code and datasets are available online at https://github.com/zhaoqi106/MPGK‐LMI.
